# Heparin versus normal saline for the care of peripheral intravenous catheters in pediatrics: a meta-analysis of randomized controlled trials

**DOI:** 10.1186/s12887-023-04515-y

**Published:** 2024-01-16

**Authors:** Ran Li, Qiaoqi Zheng, Nengyue Chen, Li Zhao

**Affiliations:** Department of Emergency, Children’s Hospital of Nanjing Medical University, Nanjing, 210000 Jiangsu Province China

**Keywords:** Heparin, Normal saline, Care, Peripheral intravenous catheter, Pediatrics, Children, Neonate, Nursing

## Abstract

**Background:**

It is still controversial for neonates or children to choose normal saline or heparin solution in the care of peripheral intravenous catheters. This meta-analysis aimed to evaluate the effects of heparin versus normal saline for the care of peripheral intravenous catheters in pediatrics, to provide reliable evidence support for clinical care.

**Methods:**

Two authors searched the PubMed, EMbase, Ovid Medline, Cochrane Library, Web of Science, CBM, WanFang Data and China National Knowledge Infrastructure (CNKI) databases for randomized controlled trial (RCT) of heparin versus normal saline for the care of peripheral intravenous catheters in pediatrics until July 16, 2023. The bias of risk tool recommended by Cochrane was used for the quality evaluation of included RCTs. Meta-analysis was carried out by using RevMan 5.4 software.

**Results:**

A total of 22 RCTs involving 3988 peripheral intravenous catheters were finally included. Compare with normal saline, heparin could significantly increase the catheter indwelling time (MD = 9.10, 95%CI:3.30 ~ 14.90). Subgroup analysis indicated that for compare with normal saline, heparin could significantly increase the catheter indwelling time in the neonate (MD = 9.63, 95%CI: 0.38 ~ 18.88) and neonate + children population (MD = 6.22, 95%CI:2.72 ~ 9.73, *P* < 0.001). Heparin could significantly reduce the incidence of catheter-associated complications (RR = 0.84, 95%CI: 0.70 ~ 0.95). Subgroup analysis indicated that heparin could significantly reduce the incidence of catheter-associated complications in the neonate (RR = 0.70, 95%CI: 0.61 ~ 0.89). There was no publication bias amongst the synthesized outcomes by Egger’s test (all *P* > 0.05).

**Conclusions:**

Heparin may be worthy of being applicated in the neonate population in terms of prolonged indwelling time and less complications. Limited by the evidence quality, more studies from different area and populations with rigorous design are needed to investigate the role of heparin versus normal saline for the care of peripheral intravenous catheters in pediatrics.

## Introduction

Peripheral intravenous catheter is the most commonly used peripheral indwelling needle in clinical nursing practice [1]. The peripheral intravenous catheter is a transfusion device with a length of 2 to 6 cm through the peripheral vein, and the end of the catheter is located in the peripheral vein [2]. Peripheral venous catheter is mainly used for clinical short-term drug infusion, but due to the uncertain direction of blood vessels in different stages of children’s growth and development, differences in puncture techniques and whether it is effective to flush and lock the catheter, it is easy to have related complications after indwelling peripheral venous catheter, such as phlebitis, drug solution exudation, catheter blockage, which eventually lead to the removal of the catheter and increase the pain of re-puncture and medical expenses [3–5]. It has been reported that the obstruction rate of peripheral intravenous catheters can be as high as 60.55% after 48 h use [6]. Once the obstruction occurs in peripheral intravenous catheters, clinical nurses will generally choose to remove it directly, which may increase the cost of medical equipment of patients [7, 8]. Therefore, the effective and safe nursing care measures for peripheral intravenous catheters are very important in clinical practice.

Currently, there is still controversy about which kind of liquid to choose for peripheral venous catheter care [9]. The commonly used clinical nursing care for lock solution is normal saline and different concentrations of heparin solution [10]. Normal saline can maintain extracellular fluid volume and osmotic pressure, which is closely related to the balance of sodium and water in the body and blood circulation. Its advantage is that the use is not limited by the type of disease. It is especially suitable for patients with bleeding tendency, disturbance of blood coagulation mechanism and insufficiency of liver and kidney [11]. Heparin sodium is a highly effective anticoagulant, it has been reported that heparin sodium can reduce venous thrombosis and maintain vascular patency [12].

At present, there are more and more studies on the lock effect of indwelling needle, but no consensus has been reached on which kind of lock solution can reduce the incidence of blockage and phlebitis and prolong the indwelling time. In the latest nursing practice guide for intravenous infusion, there is no clear recommendation on which solution (normal saline or heparin solution) for newborns or children to lock the catheter [9, 13]. Therefore, this study systematically searched the related literatures and aimed to evaluate the effects and safety of heparin versus normal saline for the care of peripheral intravenous catheters in pediatrics, to provide useful evidence for the clinical nursing care.

## Methods

This study was performed according to the preferred reporting items for systematic review and meta-analysis (PRISMA) statement [14]. Because this study was a meta-analysis, there was no need for ethical approval and patients’ informed consent.

### Inclusion and exclusion criteria

The inclusion criteria of randomized controlled trial (RCT) in this meta-analysis were: study type: RCT design. Population: Newborns to adolescents who need to indwelling peripheral venous catheters for intravenous infusion with age younger than or equal to 18 years old. Intervention: Nursing care of venous catheter sealing with heparin solution compared with 0.9% normal saline. Outcome indicators: primary outcome indicators: catheter indwelling time. Secondary outcome indicators: catheter-associated complications including phlebitis, drug extravasation and catheter blockage. The exclusion criteria for this meta-analysis were as follows: non- Chinese and English literatures; repeatedly published studies; articles that did not have access to full text or required data.

### Search strategy

We searched the PubMed, EMbase, Ovid Medline, Cochrane Library, Web of Science, CBM, WanFang Data and China National Knowledge Infrastructure (CNKI) databases for RCTs of heparin versus normal saline for the care of peripheral intravenous catheters in pediatrics until July 16, 2023. The search strategies for this meta-analysis were as following: (“peripheral intravenous catheter” OR “peripheral indwelling needle” OR “PIVC” OR “catheter”) AND (“heparin” OR “normal saline” OR “NS” OR “flushing” OR “lock”) AND (“child” OR “children” OR “pediatric” OR “neonate” OR *infant” OR “newborn” OR “adolescent” OR “young adult”). The two authors searched the database independently and then imported it to the Endnote software for further analysis.

### Literature screening and data extraction

In this meta-analysis, two evaluators independently conducted literature screening and data extraction, and cross-checked for accuracy. If there were differences, they would discuss and solve them for consensus. This meta-analysis used a pre-developed data extraction table to extract data, including: (1) the basic information included in the study, including the research topic, the name of the author, the journal published, the number of years published.; (2) the baseline characteristics of the study population, including the number of cases, gender, age, settings; (3) the specific details of the intervention measures; (4) the key elements of bias risk assessment; (5) the outcome data concerned.

### Bias risk assessment

Two researchers independently evaluated the bias risk in the study and cross-checked the results. The bias risk assessment tool recommended by Cochrane library was used for the quality evaluation of included studies [15]. The tool included seven items: random sequence generation (selection bias), allocation concealment (selection bias), blinding of participants and personnel (performance bias), blinding of outcome assessment (detection bias), incomplete outcome data (attrition bias), selective reporting (reporting bias) and other bias. Every item could be rated as “high risk of bias”, “low risk of bias” and “unclear risk of bias”.

### GRADE evidence assessment

The GRADE grading system [16] was used to evaluate the evidence quality of the outcome index, and the evidence quality was divided into four levels: high, medium, low and very low. The evidence quality grade of the outcome index was evaluated mainly from the bias of risk, inconsistency, indirectness, inaccuracy and publication bias of included RCTs.

### Statistical analysis

The meta-analysis was carried out by using RevMan 5.4 software. Mean difference (MD) or standardized mean difference (SMD) were used as effect analysis statistics, and 95% confidence interval (CI) was calculated for each effect. The heterogeneity among the included results was analyzed by χ2 test (the test level was α = 0.1). At the same time, the heterogeneity was quantitatively judged by I^2^. If there was no statistical heterogeneity among the results of each study, the fixed effect model was used for meta-analysis. If there was statistical heterogeneity among the results, the source of heterogeneity was further analyzed. After excluding the obvious clinical heterogeneity, the random effect model was used for meta-analysis. The obvious clinical heterogeneity was treated by subgroup analysis or sensitivity analysis. Publication bias was evaluated by funnel plots and Egger’s test. The significance level for all the analysis was α = 0.05.

## Results

### Literature retrieval

**Fig. 1:**
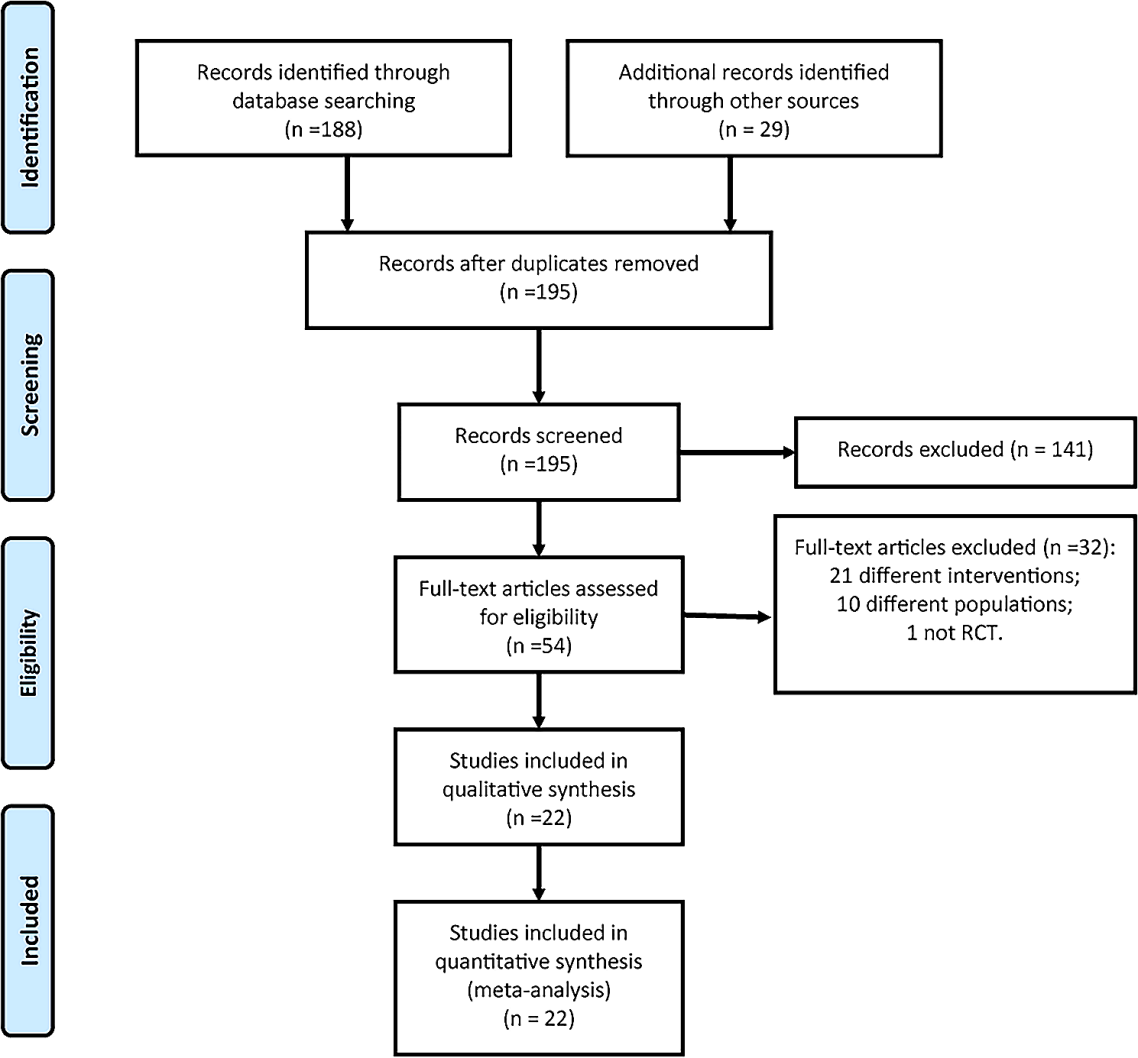
PRISMA flow diagram of RCT selection

In this study, a total of 217 related literatures were obtained in the initial search, and 22 RCTs [17–38] were finally included after layer-by-layer screening. The literature screening process and results are shown in Fig. 1.

### Characteristics of RCTs

As presented in Table 1, of the included 22 RCTs, 18 articles were reported from developed countries and regions, 4 articles from developing countries. 13 articles were published before 2000, 4 articles were published in 2000–2010, 5 articles were published in 2010 ~ 2023. The dose of heparin in RCTs varied from 0.5 to 10 U mL^− 1^, and the lock frequency of peripheral intravenous catheters remained different amongst included RCTs.

**Table 1:** Characteristics of RCTs

RCT	Country	Study population	Setting	PIVC size	PIVC	Interventions		Outcomes
						Experimental group	Control group	
Alpan 1984	Israel	Neonate	NICU	22 G	Polyvinyl chloride	Intravenous nutrition solution + heparin 1 U ·mL^− 1^	No heparin	Catheter indwelling time; complications
Beecroft 1997	USA	Neonate	NICU	22/24G	Polyvinyl chloride	NS + heparin, Q8h	NS, Q8h	Catheter indwelling time
Goldberg 1999	Canada	Neonate	NICU	24 G	Polyvinyl chloride	NS 1 mL + heparin 10 U ·mL^− 1^, Q4h	NS1 mL, Q4h	Catheter indwelling time
Heilskov 1998	USA	Neonate	NICU	24 G	Polyvinyl chloride	NS 1 mL + heparin 2 U ·mL^− 1^, Q6h	NS 1 mL, Q6h	Catheter indwelling time; complications
Inge 2011	Netherlands	Neonate	NICU	24 G	Polyvinyl chloride	NS 0.7 mL + heparin 10 U ·mL^− 1^, Q8h	NS 0.7 mL, Q8h	Catheter indwelling time; complications
John 2015	India	Neonate	NICU	24 G	Polyurethanes	NS 1 mL + heparin 10 U ·mL^− 1^	NS1 mL	Catheter indwelling time
Kleiber 1993	USA	1 ~ 18y	Department of pediatric	22 ~ 24 G	Polyvinyl chloride	NS + heparin 10 U ·mL^− 1^, Q6h	NS, Q6h	Catheter indwelling time
Klenner 2003	Germany	Neonate	NICU	24 ~ 26 G	Polyvinyl chloride	Add 1 mL NS of heparin containing 0.5U mL^− 1^ to every 100 mL infusion fluid	NS	Catheter indwelling time; complications
Kotter 1996	USA	Neonate	NICU	22 ~ 24 G	Polyvinyl chloride	NS + heparin 10 U ·mL^− 1^, Q4h	NS, Q4h	Catheter indwelling time; complications
Krista 1999	Canada	Neonate	NICU	24 G	Polyvinyl chloride	NS 1 mL + heparin 5 U ·mL^− 1^, Q6h	NS 1 mL, Q6h	Catheter indwelling time; complications
Mcmullen 1993	USA	0 ~ 18y	Department of pediatric	18 ~ 24 G	Polyvinyl chloride	NS + heparin 10 U mL^− 1^	NS	Catheter indwelling time; complications
Moclair 1995	UK	Neonate	NICU	24 G	Polyvinyl chloride	Intravenous nutrition solution + heparin 0.1/ 0.25/0.5/1 U ·mL^− 1^	No heparin	Catheter indwelling time; complications
Mok 2007	Hong Kong, China	1 ~ 10y	Department of pediatric	22/24 G	Polyvinyl chloride	NS 1 mL + heparin 1U·mL^− 1^, q6h	NS 1 mL	Catheter indwelling time; complications
Mudge 1998	USA	0 ~ 1y	PICU/NICU	24 G	Polyvinyl chloride	NS 1mL + heparin 10U·mL^− 1^	NS 1 mL	Catheter indwelling time; complications
Nelson 1998	USA	0 ~ 1y	Department of pediatric/NICU	24 G	Polyvinyl chloride	NS 1.5mL + heparin 10U·mL^− 1^, q8h	NS 1.5mL	Catheter indwelling time; complications
Paisley 1997	USA	Neonate	NICU	24 G	Polyvinyl chloride	NS 0.6 mL + heparin 10 U ·mL^− 1^, Q4h	NS 0.6 mL, Q4h	Catheter indwelling time; complications
Schultz 2002	USA	Neonate	NICU	24 G	Polyvinyl chloride	NS 0.5 mL + heparin 2 U ·mL^− 1^, Q3h	NS 0.5 mL, Q3h	Catheter indwelling time; complications
Sun 2016	China	Neonate	NICU	22 G	Polyurethanes	NS 2 mL + heparin 5 U ·mL^− 1^	NS 2 mL	Complications
Treas 1992	USA	Neonate	NICU	24 G	Polyvinyl chloride	NS + heparin 0.5 U ·mL^− 1^	NS	Catheter indwelling time; complications
Tripathi 2008	India	1 ~ 12y	Department of pediatric	22/24 G	Polyvinyl chloride	NS 1 mL + heparin 100 U ·mL^− 1^	NS 1 mL	Catheter indwelling time; complications
Upadhyay 2015	India	0.2 ~ 5y	Department of pediatric	22/24 G	Polyurethanes	NS 1 mL + heparin	NS 1 mL	Catheter indwelling time
White 2011	USA	Children	Department of pediatric	20/22/24 G	Polyurethanes	NS + heparin	NS	Complications

Notes: NICU, neonatal intensive care unit; PICU, pediatric intensive care unit; NS, normal solution

### Quality of included RCTs

**Fig. 2:**
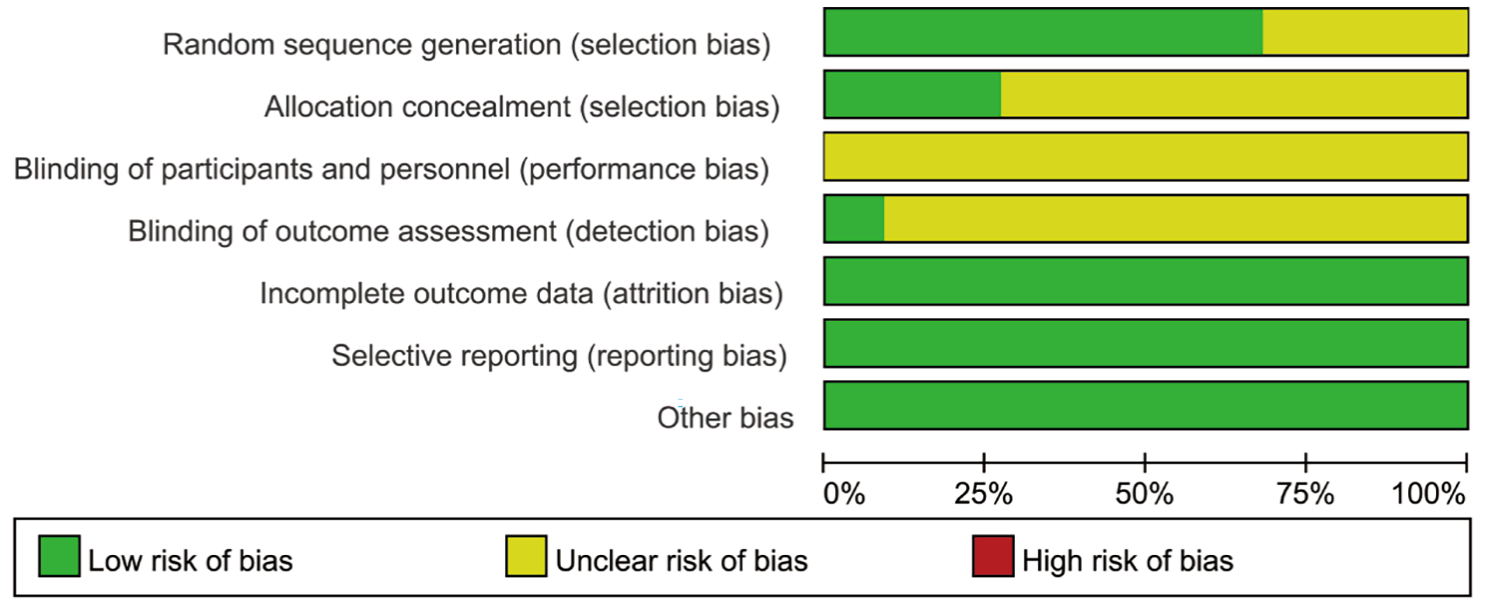
Risk of bias graph

**Fig. 3:**
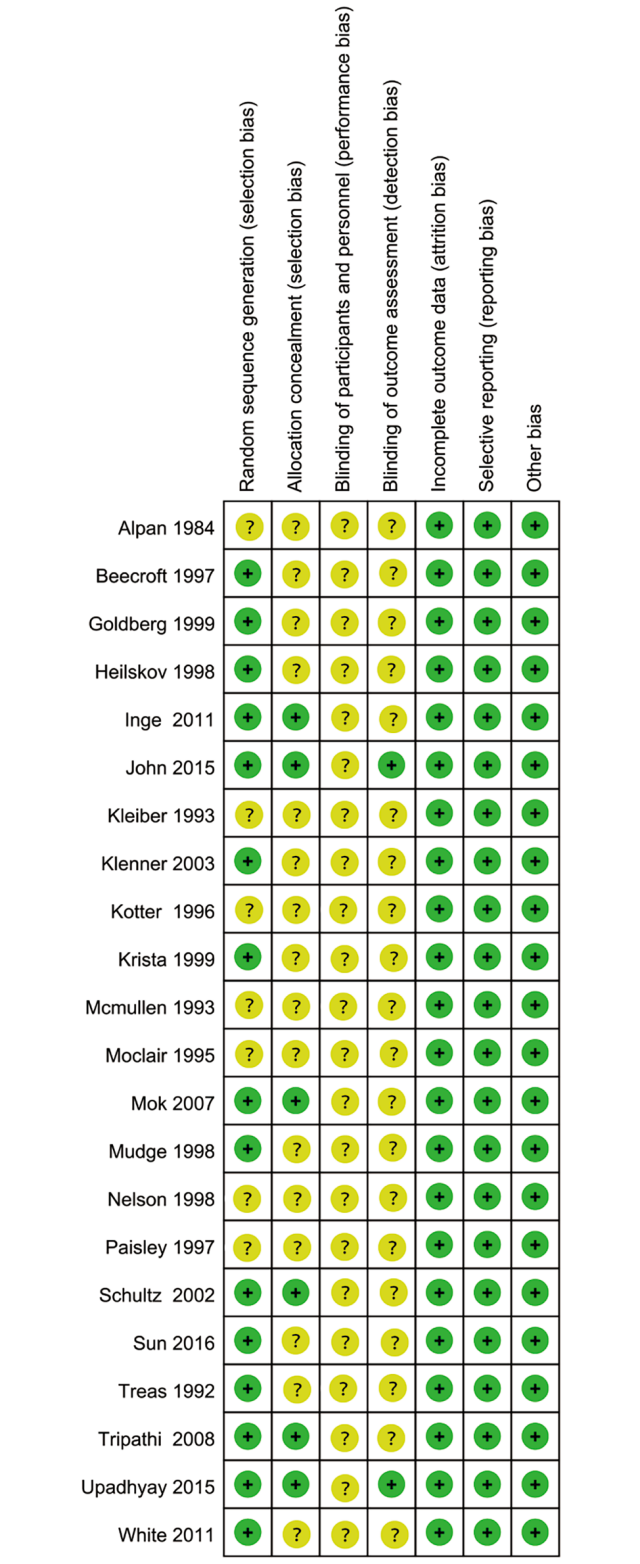
Risk of bias summary

As presented in Figs. 2, 3 and 15 RCTs reported the detailed methods for random sequence generation. 6 RCTs reported the details of allocation concealment. No reports on the blinding of participants and personnel were found. 2 RCTs reported the design on the blinding of outcome assessment, no risk of bias in the incomplete outcome data, selective reporting and other bias were found.

### Meta-analysis

**Fig. 4:**
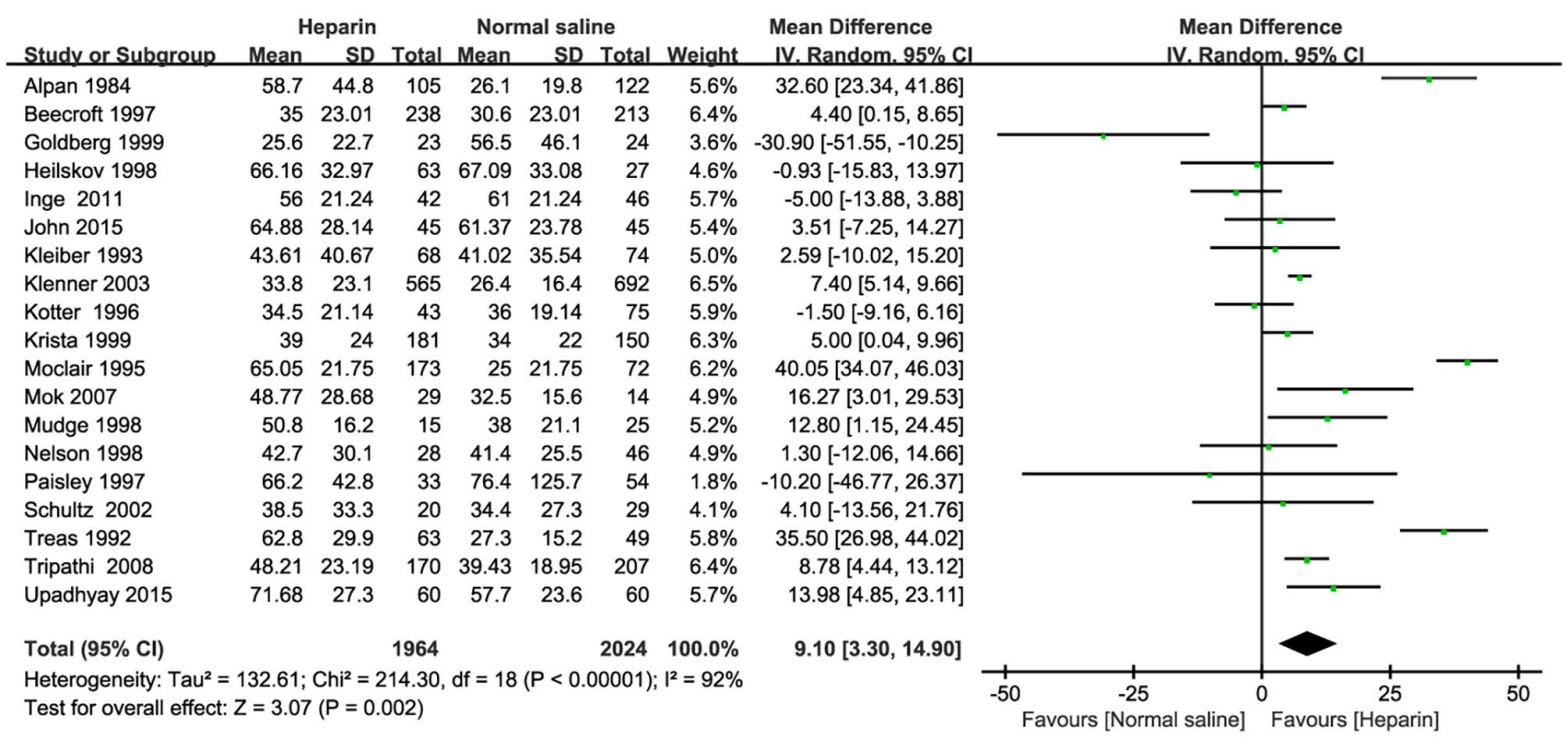
Forest plot for catheter indwelling time

19 RCTs reported the catheter indwelling time. As shown in Fig. 4, meta-analysis indicated that compared with normal saline, heparin could significantly increase the catheter indwelling time (MD = 9.10, 95%CI:3.30 ~ 14.90, *P* = 0.002). As presented in Table 2, subgroup analysis indicated that for compare with normal saline, heparin could significantly increase the catheter indwelling time in the neonate (MD = 9.63, 95%CI:0.38 ~ 18.88, *P* = 0.042) and neonate + children population(MD = 6.22, 95%CI:2.72 ~ 9.73, *P* < 0.001), no effect difference in the catheter indwelling time in the children population(MD = 6.94, 95%CI: -1.27 ~ 15.15, *P* = 0.100) were found.

**Table 2:** The subgroup analysis on the catheter indwelling time

Variable	Number of included RCTs	Heterogeneity (I^2^)	Model for meta-analysis	Mean difference	95%CI	*P*
Neonate	12	95%	Random	9.63	0.38 ~ 18.88	0.042
Children	3	27%	Fixed	6.94	-1.27 ~ 15.15	0.100
Neonate + children	5	20%	Fixed	6.22	2.72 ~ 9.73	< 0.001

19 RCTs reported the incidence of catheter-associated complications. As shown in Table 3, meta-analysis indicated that compare with normal saline, heparin could significantly reduce the incidence of catheter-associated complications (RR = 0.84, 95%CI: 0.70 ~ 0.95, *P* = 0.002). Subgroup analysis indicated that for compare with normal saline, heparin could significantly reduce the incidence of catheter-associated complications in the neonate (RR = 0.70, 95%CI: 0.61 ~ 0.89, *P* = 0.004). No effect differences in the catheter indwelling time in the children population (RR = 0.94, 95%CI: 0.62 ~ 1.41, *P* = 0.751) and neonate + children population (RR = 0.98, 95%CI: 0.71 ~ 1.33, *P* = 0.904) were found.

**Table 3:** The meta-analysis and subgroup analysis on the incidence of catheter-associated complications

Variable	Number of included RCTs	Heterogeneity(I^2^)	Model for meta-analysis	Risk ratio	95%CI	*P*
Total	19	11%	Fixed	0.84	0.70 ~ 0.95	0.002
Neonate	10	5%	Fixed	0.70	0.61 ~ 0.89	0.004
Children	4	19%	Fixed	0.94	0.62 ~ 1.41	0.751
Neonate + children	5	20%	Fixed	0.98	0.71 ~ 1.33	0.904

This study used the method of removing individual studies one by one for sensitivity analysis, the results showed that there was no significant change, suggesting that the results of this study were stable.

**Fig. 5:**
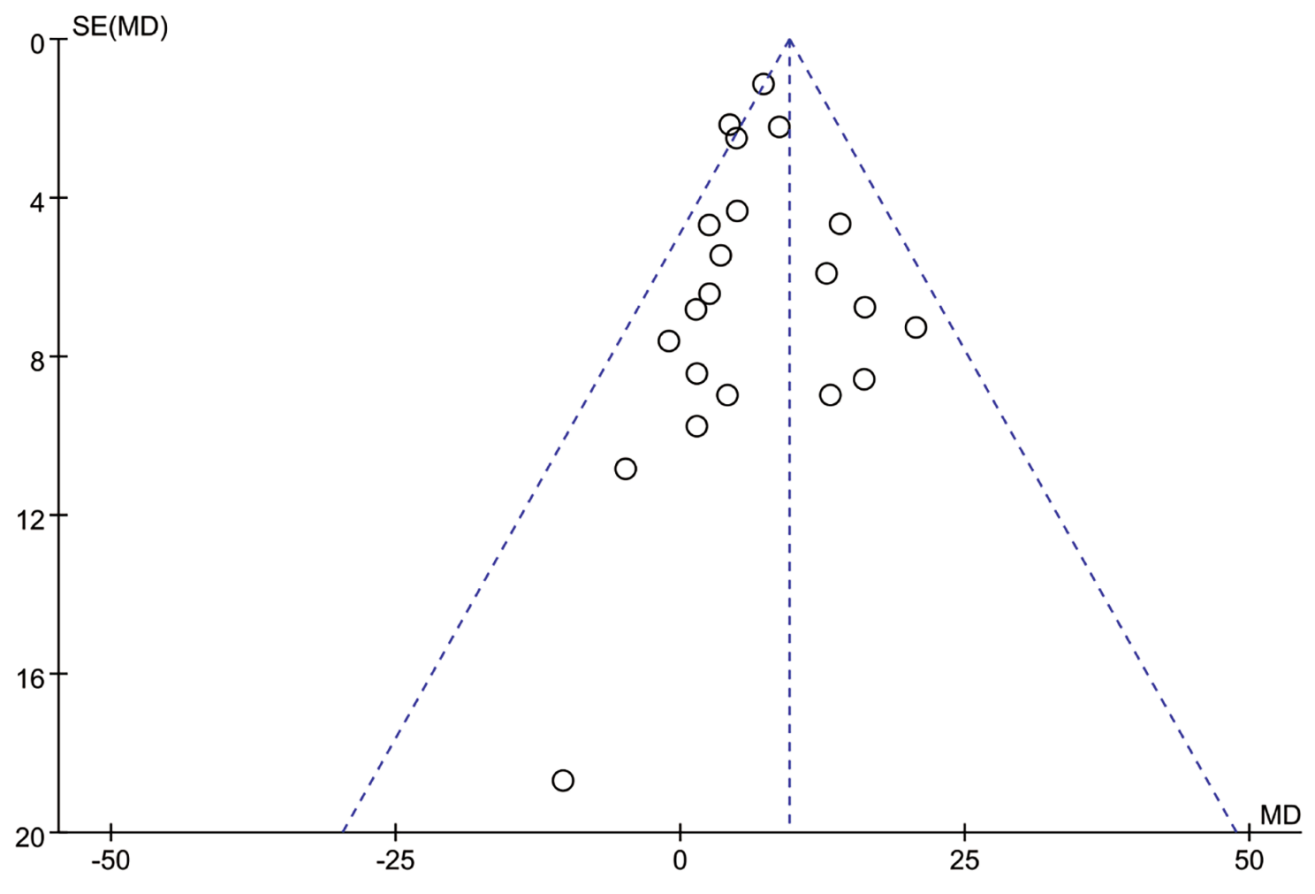
Funnel plot for catheter indwelling time

The funnel plot (Fig. 5) and results of Egger’s test of publication bias showed that the possibility of publication bias was small (All *P* > 0.05).

### Evidence quality

As indicated in Table 4, the evidence on the catheter indwelling time was in middle level, and the evidence on the incidence of catheter-associated complications was in low level.

**Table 4:** Evidence quality classification of outcome indicators

Outcomes	Risk of bias	Inconsistency	Indirectness	Inaccuracy	Publication bias	Evidence quality
The catheter indwelling time	Small	No	No	No	No	Medium
The incidence of catheter-associated complications	Small	No	Yes	Yes	No	Low

## Discussions

Peripheral intravenous catheter is one of the most commonly used venous pathways in clinical practice. At present, the clinical lock solution for peripheral intravenous catheters has a portable solution of 0.9% normal saline, which can greatly reduce the workload and working time of clinical nurses, but the lock solution of peripheral intravenous catheters is also a controversial topic in clinical care [39, 40]. The previous systematic review [41] has shown that heparin cannot prolong the use of peripheral intravenous catheter compared with normal saline. However, the other systematic review [40] has reported that the use of heparin is beneficial to significantly prolong the use of peripheral intravenous catheters and reduce the incidence of complications. The above completely different conclusions may be related to the number of literatures included and the different concentrations of heparin infusion. With more RCTs included, the results of this meta-analysis have found that heparin have more advantages over normal saline for the care of peripheral intravenous catheter in indwelling time and the incidence of catheter-associated complications. Heparin may be more appropriate for the clinical care practice of peripheral intravenous catheters in pediatrics.

The application of peripheral intravenous catheters greatly satisfies the patients who need short-term infusion. Heparin sodium is a kind of acidic mucopolysaccharide and has strong anticoagulant effect both in vivo and in vitro [42]. Therefore, it can effectively reduce the blood flowing back into the indwelling needle to form blood clots and block the pipeline, which is widely used in clinical care. Sealing the tube with heparin can effectively reduce the incidence of blockage and shorten the time of blockage, so as to reduce the replacement of patients due to the blockage of indwelling needle, reduce the pain of puncture, and achieve the saving of medical resources in a certain range [43–45].

It must be noted that two included RCTs have reported that intracranial hemorrhage is associated with the use of heparin. There is no significant difference in the incidence of intracranial hemorrhage between heparin and saline, but it still needs clinical attention. Besides, two included RCTs reported the occurrence of thrombocytopenia induced by heparin. Although there is no significant difference in thrombocytopenia induced by heparin the between heparin and normal saline, it was still necessary to detect the corresponding clinical indexes when using heparin in children with contraindications of heparin. Some scholars [46, 47] have reported that normal saline is safer than heparin sodium in patients with cardiovascular diseases, gastrointestinal bleeding and hematological diseases. Therefore, the lock solution can be used reasonably according to the specific conditions of the pediatrics [48].

There are several limitations of this study must be considered. Firstly, the RCTs included in this meta-analysis have been published for a relatively long time. With the development of materials and technology, there may be some differences in the quality and design of peripheral intravenous catheters. Secondly, there are very high statistical heterogeneity (I^2^ = 92%) in the synthesized outcome, which may be related to the wide age range of the participants, the different underlying diseases whose treatments might interfere with coagulation, the different characteristics of the prescriptions, the frequency of use, and the very different concentrations of heparin sodium. Most of the RCTs reports included in this meta-analysis are from developed countries, and there is still a lack of relevant report data from developing countries. Future studies with larger sample size from different area and populations are needed. Finally, most of the RCT studies included in this paper do not mention blind setting and allocation concealment. It is suggested that future studies should further improve the RCT design.

## Conclusions

In summary, in the selection of lock solution of peripheral intravenous catheters in children, heparin saline can effectively prolong the indwelling time of peripheral intravenous catheters and reduce the incidence of related complications than normal saline. However, the evidence quality is not high, the findings should be treated with cautions. Under the circumstances of the shortage of medical resources and human resources of pediatric nurses, heparin may be recommended to the care of peripheral intravenous catheters in pediatrics when the children do not have blood coagulation dysfunction in clinical nursing care, which may effectively prolong the use of indwelling catheter and reduce the pain caused by repeated puncture.

## Data Availability

All data generated or analyzed during this study are included in this published article. The original data will be available from corresponding authors on reasonable request.

## Abbreviations

PRISMA: Preferred reporting items for systematic review and meta-analysis
RCT: Randomized controlled trial
NICU: Neonatal intensive care unit
PICU: Pediatric intensive care unit
MD: Mean difference
SMD: Standardized mean difference
CI: Confidence interval
